# How trust shapes individual resilience to natural hazards: a systematic review

**DOI:** 10.1007/s11069-025-07816-w

**Published:** 2026-01-19

**Authors:** Joshua P. Nicholas, Amy Donovan, Clive Oppenheimer, Louie Bell, Maximillian Van Wyk de Vries

**Affiliations:** 1 Department of Geography, University of Cambridge, 20 Downing Place, Cambridge, CB2 1DB UK; 2 Department of Earth Sciences, University of Cambridge, Downing Street, Cambridge, CB2 3EQ UK

**Keywords:** Trust, Resilience, Natural hazards, Disaster risk reduction, Vulnerability, Risk perception

## Abstract

Trust is recognised as a critical element of disaster risk reduction; it shapes how people perceive risks, mobilise resources, and respond to hazards. Here, trust is defined as an individual’s confidence in the ability and/ or intention of a system to act in their best interest. Importantly, many studies do not distinguish between ability- and intention-trust or acknowledge that such a distinction exists. Here, we present a systematic review of 100 articles and book chapters published between 2000 and 2025 which reveals that trust can both strengthen and weaken resilience to hazard events, depending on geographical factors, cultural context, hazard type, and the parties in whom trust is placed (e.g., government, community, scientific institutions, or personal beliefs). From the 100 studies, we identified 209 relationships between trust and individuals’ resilience to natural hazards. We find that in the majority of case studies, trust is associated with increased resilience (58%), compared with trust being associated with decreased resilience (33%), or no change to resilience (10%). Our findings highlight the need for clarity when defining or theorising trust, and recognise the dynamic and context-dependent nature of trust when seeking to improve resilience to support effective disaster risk reduction.

**Supplementary Information:**

The online version contains supplementary material available at 10.1007/s11069-025-07816-w.

## Introduction

Trust has emerged as a critical element in disaster risk reduction (DRR), particularly with respect to risk perceptions, social vulnerability, and resilience (Wachinger et al. 2013; Bonfanti et al. 2023; Cisternas et al. 2024). Trust is integral to daily life. In areas where we lack sufficient knowledge, we rely upon the knowledge or expertise of others (Seebauer and Babcicky 2018; Dias et al. 2020). From trusting chefs not to poison our food, to trusting pilots to know how to fly, trust is ubiquitous. Trust lacks a uniform definition within DRR. Some use it to represent an individual’s confidence that a system has the *ability* to undertake a specific action (Martinez et al. 2020; Asfaw et al. 2022). Alternatively, it may refer to confidence that a system has the *intention* to undertake a specific action (Reininger et al. 2013; Wong et al. 2021). Some works recognise a combination of these two perspectives (Seebauer and Babcicky 2018; Moreno et al. 2019) while others make no specification (Bird et al. 2017; Kerstholt et al. 2017). Empirical findings on the effect of trust vary across cultural and social contexts, highlighting that trust functions differently in different settings (Seebauer and Babcicky 2018; Bonfanti et al. 2023).

In much of the DRR literature, resilience is framed as the ultimate goal for individuals, communities, and nations seeking to prepare for, mitigate against, and recover from natural hazards (Wisner et al. 2003; Alexander 2013). In DRR, resilience is a central goal amid rising climate-related losses (Di Baldassarre et al. 2018; Ritchie and Rosado 2023). Studies link resilience to preparedness, mitigation, evacuation, social support, and sometimes risk perception, while debating its definition and various determinants (e.g., demography, exposure, experience, and trust) (Cutter et al. 2003; Terpstra 2011; Cutter 2016; Kelman et al. 2016).

This rising interest in “resilience” coincides with rising economic losses from hazard events over the past two decades, which in part reflect increasing frequency of hydrometeorological disasters under climate change (Di Baldassarre et al. 2018; Ritchie and Rosado 2023). Within the context of natural hazards, resilience is frequently linked to preparedness, mental health, evacuation decisions, social support, and at times risk perception (Terpstra 2011; Moreno et al. 2019; Babcicky and Seebauer 2020; Bonfanti et al. 2023). However, the term remains contested across context and scales (Adger 2000; Cutter et al. 2003; Cutter 2016; Kelman et al. 2016), reflecting the diversity of demographics, socioeconomics, exposure, living conditions, hazard experience, and the nature and extent of trust (Cutter et al. 2003; Gaillard 2010; Terpstra 2011).

Here, we systematically review research published between 2000 and 2025 to explore how trust shapes natural hazard resilience. While Bonfanti et al. (2023) addresses the role trust plays in disaster prevention, preparedness, response, and recovery stages, our analysis differs by examining how different methodologies, geographic locations, and definitions of trust affect its relationships with resilience. Our specific aims are to:

i. Assess the varied conceptualisations of “trust” and “resilience” within the DRR literature.
ii. Synthesise the state of research, highlighting key patterns in how trust is measured and how it may support or undermine resilience.

## Background

### Vulnerability and resilience in disaster risk reduction

The concept of “resilience” in hazard research emerged partially from Holling’s (1973) ecological systems work, which defined resilience as a system’s capacity to absorb disturbances without losing its core functions (Holling 1973). DRR literature then adapted this definition to social systems as shown when in 2004 the United Nations (UN) defined resilience as the capacity of a system to “resist or change” to maintain functionalities (UNISDR 2004). As definitions of “resilience” have evolved over the past two decades, scholars have debated whether resilience should emphasise returning to a pre-disaster norm (i.e., resisting the effects of hazards) or to “bounce forward” to a more sustainable state (Christoplos 2006; Manyena 2006; Gaillard 2010). The latter is increasingly becoming the standard, as exemplified by recent European recovery scholarship that emphasises how reconstruction policy design and relocation choices mediate “build back better” trajectories following extreme floods (Birkmann et al. 2023; Truedinger et al. 2023). The Hyogo Framework for Action and the Sendai Framework for Disaster Risk Reduction have cemented “resilience” as a key goal in DRR (UNISDR 2005, 2015). However, despite its popularity within DRR, scholars are not all in agreement on its definition. Some frame resilience as inversely related to vulnerability, while others argue they can coexist (Kelman et al. 2016). Another critique is that governments might place the onus of “being resilient” on communities, absolving higher-level entities of responsibility (Mikulewicz 2019).

While there is some agreement that “resilience” is relevant to both the pre- and post-disaster phases through mitigation, preparation, response, and recovery, its lack of a clear definition still leads to criticism and confusion (Cutter et al. 2008; Alexander 2013; Moreno et al. 2019). Resilience research often focuses on individuals’ preparedness and mitigation, though there is growing interest in how communities respond to and recover from disasters; this highlights the importance of considering all phases of disasters in resilience research (Bonfanti et al. 2023). Moreover, resilience can manifest at multiple scales (e.g., organisational, community, ecosystem) and can depend heavily on cultural, political, and economic contexts (Nicholls and Picou 2013; Han et al. 2021). In practice, prevention and mitigation hinge on how spatial planning and risk governance align across agencies, with stakeholder participation shaping the feasibility and acceptance of structural and non-structural measures (Sapountzaki et al. 2011; Fleischhauer et al. 2012).

### Defining trust

The word “trust” has taken an interesting etymological journey partially shared with the words “true” and “tree”. While the origins are debated, the word is believed to be traceable back to the Proto Indo-European word *deru-* meaning to “be firm, solid, steadfast” (Etymology Dictionary 2024). This history of “trust” referring to the strength of good faith and confidence in a person or thing has evolved into its present meaning: a psychological state formed of the intention to accept vulnerability in exchange for expected positive intentions or actions from someone or something else (Rousseau et al. 1998). This definition emphasises that willing acceptance of vulnerability by the trusting party is key to the concept of trust, even without guarantees regarding the trusted party’s motivations (Wei et al. 2019).

**Fig. 1:**
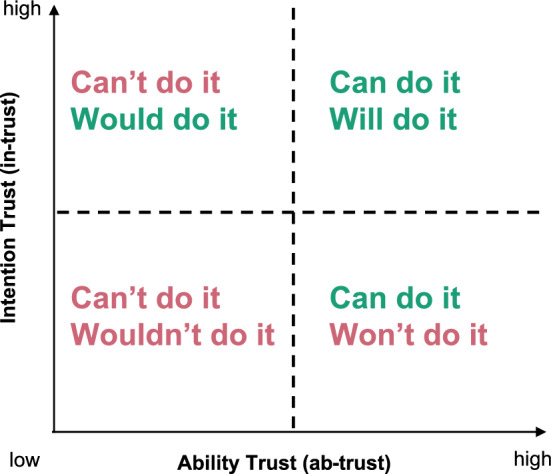
Relationship between ability and intention trust in an institution/ person from the perspective of what an individual believes an institution/ person can or would do

Researchers distinguish among different objects of trust; some focus on intention trust (i.e., confidence in the motivations and goodwill of a person or institution) while others highlight ability trust (i.e., confidence in the competence and capability of a person or institution) (Eiser et al. 2012; Seebauer and Babcicky 2018; Houston et al. 2019). In practice, many studies combine these definitions or do not explicitly differentiate between the two. In Fig. 1 we illustrate how ability and intention trust interact. The horizontal axis (low to high) represents ability trust (“ability‐trust”), or whether one believes that a person or institution *can* do something; the vertical axis (low to high) represents intention trust (“intention‐trust”), or whether one believes that a person or institution *will* do something. Thus, in the top‐right quadrant (high intention and high ability) an individual perceives the institution as both able and willing to help. By contrast, the bottom‐right quadrant shows an institution seen as capable but unwilling, while the top‐left quadrant shows an institution seen as willing but not capable, and the bottom‐left reflects doubt in both its willingness and its capability.

### Trust, resilience, and social capital

Multiple theoretical lenses help explain when, how, and why trust relates to resilience in natural hazard contexts. Here we draw on risk perception theories, social capital theory, and recreancy theory to clarify behavioural pathways linking trust to preparedness, mitigation and prevention uptake, response, and recovery.

Risk perception theories, including appraisal-based accounts, posit that protective action depends on how people evaluate threat and coping options, and on the credibility of information and messengers (Terpstra 2011; Eiser et al. 2012). Trust shapes these appraisals by affecting whether warnings are believed, whether recommended measures are seen as feasible and effective, and whether institutions are viewed as competent and benevolent. Trust is associated with the well-documented “risk perception paradox” (i.e., high concern with limited action) as the paradox reflects mismatches between trust in authorities versus peers, doubts about institutional capacity, and low perceived efficacy despite acknowledged risk (Wachinger et al. 2013).

Many researchers discuss trust as a component or indicator of social capital, which comprises the networks, norms, and relationships enabling communities to act collectively (Putnam 1995; Bourdieu 2018). Social capital can be critical during disasters by facilitating cooperation, resource-sharing, and collective efficacy (Reininger et al. 2013; Castro-Correa et al. 2020). Social capital is not reducible to trust, and high trust can sometimes dampen protective action; for example, where reliance on familiar networks reduces perceived urgency or fosters local norms that prioritise staying put over evacuation, underscoring that social capital’s effects are context specific (Minamoto 2010; Story et al. 2020). While high levels of trust and social capital often correlate with better community preparedness and recovery (Bonfanti et al. 2023), these correlations are highly geographically dependent.

Recreancy theory foregrounds public judgments about whether institutions discharge their fiduciary responsibilities; that is, whether they are technically competent and act in the public interest when managing risks (Freudenburg 1993; Tierney 2012, 2025). It therefore speaks directly to ability-trust (perceived capability) and intention-trust (perceived intent), and adds explicit attention to responsibility, blame, and accountability. As summarised by Ritchie (2024), recreancy encompasses concerns about potential harm, responsibility for disaster impacts, confidence in institutional competence and intent, denial of responsibility, disinformation, post-disaster compensation, and institutional performance in judicial redress (Ritchie 2024). Although often applied to technological risks, the same logic applies to natural hazards, where perceived failures in planning, warnings, infrastructure maintenance, or recovery processes can trigger recreancy judgments that suppress compliance, reduce uptake of mitigation and relocation, and erode cooperation. Conversely, visible competence, procedural fairness, and timely compensation can restore trust and enable “build back better” trajectories. In this view, people act not only on perceived danger but on whether responsible institutions are seen as both able and willing to meet their obligations.

Cultural theory (Douglas and Wildavsky 1983) and its contemporary extension, cultural cognition (Kahan and Braman 2006), argue that shared cultural worldviews shape who is deemed credible and which risk responses feel legitimate. Grid-group orientations (hierarchist, individualist, egalitarian, fatalist) and culturally congruent identities guide trust in particular messengers (authorities, peers, faith leaders) and preferred solutions (infrastructure, self-reliance, communal action), thereby conditioning both ability and intention trust. This framework can help explain culturally specific responses. For example, evacuation, which is often seen as a resilience indicator, may not always be deemed appropriate, especially in communities that prioritise sheltering in place or protecting properties (Asfaw et al. 2022). Similarly, some cultures place higher trust in spiritual beliefs, potentially leading to lowered perceived risk (Salah and Sasaki 2021). Resilience research must carefully account for differing hazard contexts and cultural practices.

## Methods

**Fig. 2:**
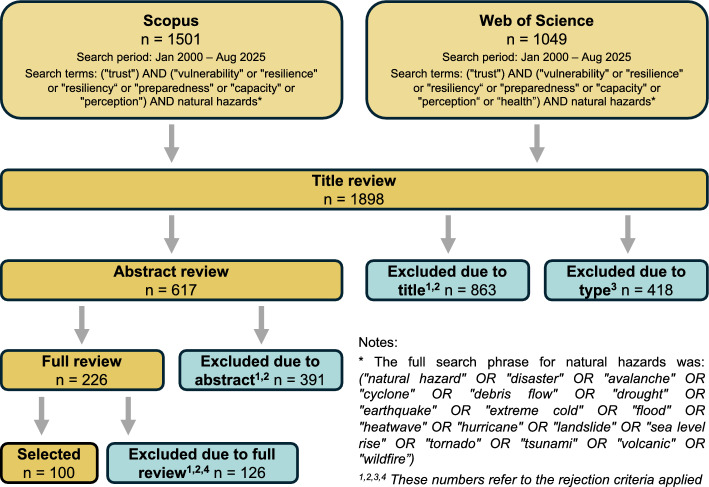
Summary of data collection method highlighting the number of articles accepted and rejected at each stage of the selection process

We performed a systematic literature review of articles relating to trust in the context of resilience to natural hazards (Fig. 2). We used the Scopus and Web of Science literature databases to search for relevant articles published between January 2000 and August 2025; this timeframe was chosen to reflect the growing momentum of resilience research in the early 2000s (Gunderson 2000; Cutter et al. 2003; UNISDR 2005). The general structure of the Boolean search in both databases was as follows:


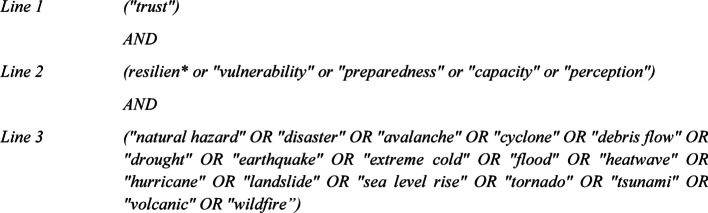


These search terms were applied to the title, abstract, and keywords of articles in each database to identify relevant studies. While “trust” is strongly associated with “confidence”, we decided to include only the term “trust” in *Line 1*; this was to ensure we could compare and contrast definitions of “trust” between the articles reviewed. *Line 2* of the Boolean search included the term “vulnerability” due to its close relationship with resilience (Kelman et al. 2016). The terms “preparedness”, “capacity”, and “perception” were included as they are referenced as critical components of vulnerability and resilience (Rufat et al. 2015; Drakes and Tate 2022). To ensure that papers referencing a specific hazard without also using the term “natural hazard” were included in the search, *Line 3* includes a list of common hazard types; this list was formed using common terminology and hazard types in the DRR literature (Berz et al. 2001; UNISDR 2009; Gill and Malamud 2014).

Following these criteria, we identified 1501 and 1049 relevant articles in the Scopus and Web of Science databases respectively; 652 articles were identified in both databases leading to a total of 1898 unique articles. To identify appropriate papers for this study, we established the following five exclusion criteria:

1. The study does not consider the combination of trust, resilience, and natural hazards.
2. The study considers terrorism, warfare, chemical, biological/ medical, or technological hazards.
3. The study does not report at least one explicit relationship between trust (or distrust) and a resilience outcome.
4. The article is in the form of a conference paper, review article, editorial, proceedings paper, or book.
5. The article was not available to us for PDF download online.

**Fig. 3:**
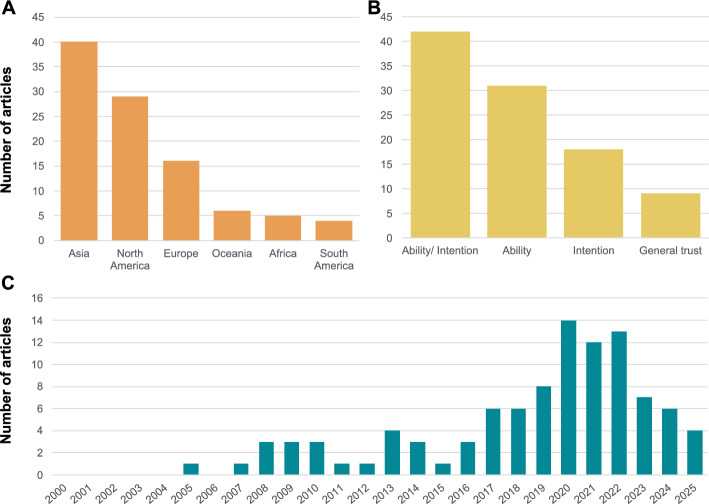
**A** Number of selected articles per continent. **B** Number of selected articles per measure of trust. **C** Number of selected articles per year of publication within the review timeframe (2000–2025)

Using these criteria, JPN and LB independently reviewed the titles of all 1898 papers to form a subset of 617 papers whose abstracts were reviewed yielding 226 papers that were then subjected to a full review by JPN. Of these, 110 articles were excluded (due to lack of relevance or lack of measurable findings on the relationship between trust and resilience) and a further 16 were not available for download, leaving 100 articles and book chapters for analysis. Because included studies were conceptually and methodologically heterogeneous and a meta-analysis was inappropriate, we followed SWiM guidance for transparent “synthesis without meta-analysis” (Campbell et al. 2020). While our approach excluded grey literature and publications in languages other than English, the 100 studies span 32 countries and territories, offering broad geographical representation. Asia represented the highest number of case studies (40), followed by North America (29), Europe (16), Oceania (5), and South America (4). Most of the studies were published since the beginning of 2020 (Fig. 3).

Each article was reviewed to extract variables representing hazard type, research methods, definitions and measures of trust and resilience, epistemology, sample size, and location. We used frequency tables to identify patterns in these variables between the articles (Rufat et al. 2015; Drakes and Tate 2022). Thematic analysis was then carried out using NVivo, employing inductive coding of methods, results, and discussion sections. We adopted direction of effect as the standardised metric for findings (increase/decrease/mixed or no effect on the specified resilience outcome). We recognise that trust and distrust are not simple opposites conceptually, and we do not assume psychological symmetry between them. However, to place findings on a common direction-of-effect scale for synthesis where a study reported distrust, we inverted the sign (e.g., “distrust in government increases risk perception” is recorded as “trust in government decreases risk perception”). This recoding is an analytic convenience only to align arrows of effect and does not imply equivalence of constructs; empirically in our sample, studies using distrust measures generally showed inverse associations to analogous trust measures. Conducting both a variable analysis and thematic analysis we sought to capture both quantitative trends and deeper qualitative insights of how trust may shape natural hazard resilience. When summarising headline findings in text and figures, we prioritised results that (i) directly measured trust against a resilience outcome, (ii) used larger samples or designs closely matching the review aims.

## Results

This section presents key findings from 100 studies investigating how trust influences natural hazard resilience. The reviewed works span multiple regions and employ varying methodologies. The review finds that trust (which is itself defined and measured in diverse ways) can both strengthen and weaken resilience, depending on various factors including cultural context, hazard type, and the institutions involved.

### Understanding trust and resilience

While most authors do not define their measure of trust, based on our review of the methods used, we labelled each study as measuring intention trust (18%), ability trust (32%), a mix of both (42%), or neither (i.e., measuring general trust) (9%). Research that focuses on ability trust tends to examine the levels of trust in a system’s capacity to prevent, manage, or mitigate disasters. Meanwhile, research into intention trust encompasses trust in the motives, reliability, and commitment of the trusted party to act in the trustor's interest. Example questions for both types of trust are outlined in Table S1 in (see Supplementary material 1).

Importantly, differences arise in how trust is defined. For Sharp et al. (2013) trust is described as a willingness to rely on another based on the expectation of action or intention to act in a beneficial manner. This definition is expanded by Schwaller et al. (2021) who regard trust as a dynamic of four key dimensions: competence, commitment, caring, and predictability. Some researchers distinguish between trust and trustworthiness where ‘trustworthiness’ represents a perceived characteristic of a party reflecting their ability, intention, and integrity (Sharp et al. 2013). Trust is also frequently cited as a component in that it enhances social cohesion and collective efficacy (Reininger et al. 2013; Hernández Aguilar and Ruiz Rivera 2016; Castro-Correa et al. 2020).

**Fig. 4:**
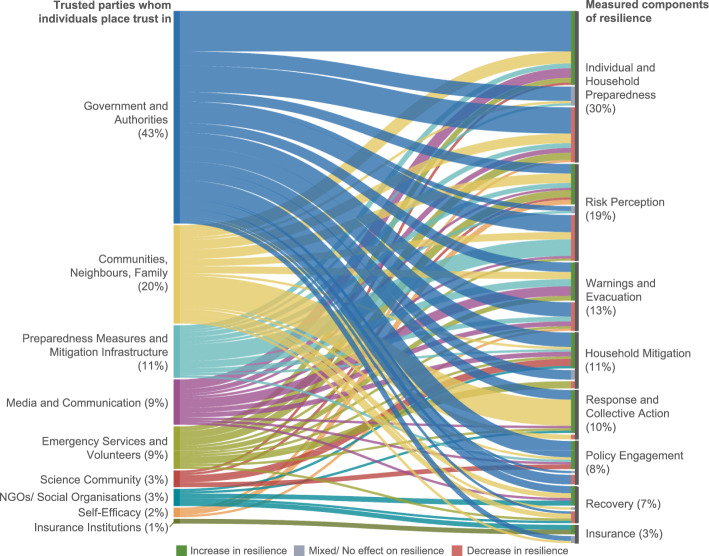
Alluvial diagram showing on the left the nine “trusted parties” people place trust in, and on the right, the eight measured components of resilience. The diagram shows the percentage of trust-resilience relationships identified for each trusted party and each resilience component. The diagram illustrates the effect that trusting in a given party has on a specific resilience component (i.e., increase, decrease, or no effect). Both the trusted parties and resilience components are listed in decreasing frequency of identified relationships (e.g., 43% of the 209 trust-resilience relationships considered government & authorities as the “trusted party” while 19% of the 209 trust-resilience relationships measured risk perception as the resilience metric) (made using Flourish.studio)

The reviewed studies primarily focus on at-risk individuals as the trusting party, though some examine specific groups such as farmers (Ma et al. 2022; Nguyen-Trung et al. 2024) and foreign residents and immigrants (Cadwell 2019; Yong et al. 2020). There is more diversity in the number of trusted parties that researchers consider, which can be categorised as follows (Fig. 4; see Supplementary material 2).

1. Government and Authorities—Includes local, state, and federal governments, officials, and public bodies responsible for orders, warnings, policy and programmes excluding science agencies (see “Science Community”).
2. Community, Neighbours, Family—Considers the community as a whole, including neighbours, family members, community leaders, businesses, local organisations, strangers, Indigenous/ local knowledge holders, and peer networks.
3. Media and Communication—Refers to official and unofficial information-dissemination outlets including TV and radio, weather reports, social media, disaster warning systems, and translators.
4. Science Community—Includes engineers, scientists, and government science agencies, as well as hazard experts and disaster managers working alongside scientific advisory teams.
5. Preparedness Measures and Mitigation Infrastructure—Refers to physical structures and tools (e.g., levees, flood-control works, shelters, pumps/hoses/sprinklers, “concrete countermeasures”) and non-physical arrangements (e.g., evacuation plans and other mitigation programmes).
6. Emergency Services and Volunteers—Considers official emergency response organisations (e.g., fire management agencies, local fire departments, civil protection, EMS) and volunteer-run or spontaneous volunteer groups involved in relief and coordination.
7. Personal Beliefs/Self-Efficacy—Refers to personal knowledge and confidence in one’s ability to manage risk (e.g., prepare, defend, evacuate), shaping response- and self-efficacy.
8. NGOs and Social Organisations—Includes non-governmental and community-based organisations, charities and faith groups, and other non-state service providers that bridge between communities and authorities and support relief, advocacy and implementation.
9. Insurance Institutions—Includes institutions that provide natural hazard-specific insurance to homeowners, businesses, and farms.

When it comes to assessing resilience, it was noted that not all articles explicitly used the term “resilience”; some framed their analysis in terms of vulnerability, social capital, or preparedness (Hernández Aguilar and Ruiz Rivera 2016; Bixler et al. 2021). To synthesise findings, we grouped resilience indicators into eight interdependent factors frequently highlighted in DRR (Table 1).

**Table 1:** Key factors of resilience as identified in the reviewed articles

Resilience factor	Sub-components	Example references
Risk perception	Perceived probability and consequences	Terpstra (2011), Seebauer and Babcicky (2018)
	Degree of confidence versus dread/ worry	
	Sense of vulnerability/hazard concern	
	Spatial awareness of hazards	
Warnings and evacuation	Acceptance of hazard warnings	Sherman-Morris (2005), Lynch et al. (2024)
	Likelihood of acting on evacuation orders	
	Time taken to comply with evacuation orders	
	Likelihood of being searched for in emergency scenarios	
Individual and household preparedness	Number and type of preparedness measures taken or intended to take (measured by plan-making, evacuation drills, escape planning, key preparedness purchases, total preparedness action counts)	Choi and Wehde (2020), Brata et al. (2024)
	Acceptance of risk management responsibilities	
Response and collective action	Efficacy of community engagement and social support	Kilby (2008), Castro-Correa et al. (2020)
	Perception of community resilience	
Recovery	Time taken to achieve recovery/expected time to recover	McCormack and Sillick (2017), Hsueh (2019)
	Degree of psychosocial recovery post-trauma	
	Long-term cooperation and collective action	
	Long-term well-being	
Policy engagement	Interest in or adoption of resilience policies	Huss et al. (2012), Ghasemi et al. (2020)
	Participation in buyouts/relocation/cooperatives/acceptance/implementation of DRR measures	
	Support for safety policy and programmes	
Household mitigation	Physical measures to reduce the risk of losses (e.g., fire-resistant siding, sprinklers, firewood clearance, private flood-protection, pumps/hoses/sprinklers)	Vignaroli (2017), Briccetti et al. (2025)
Insurance	Uptake (or intended uptake) of hazard-related insurance policies	Reynaud et al. (2018), Zinda et al. (2021)

Given the varied components of resilience considered in these studies, there is no standard for how resilience was regarded. For quantitative studies, we examined cases of statistically significant increases, decreases, or lack of changes to the sub-components of each resilience factor as a result of trust. For qualitative studies we considered relationships presented in the results sections of the reviewed papers. We identified 209 relationships between trust and resilience where trust was reported to increase, decrease, or have a mixed or null effect on individuals’ natural hazard resilience. Of these, the majority of relationships indicate that trust is associated with an increase in natural hazard resilience (58%, n = 121), compared with trust decreasing resilience (33%, n = 68), or having a mixed or null effect (10%, n = 20).

**Fig. 5:**
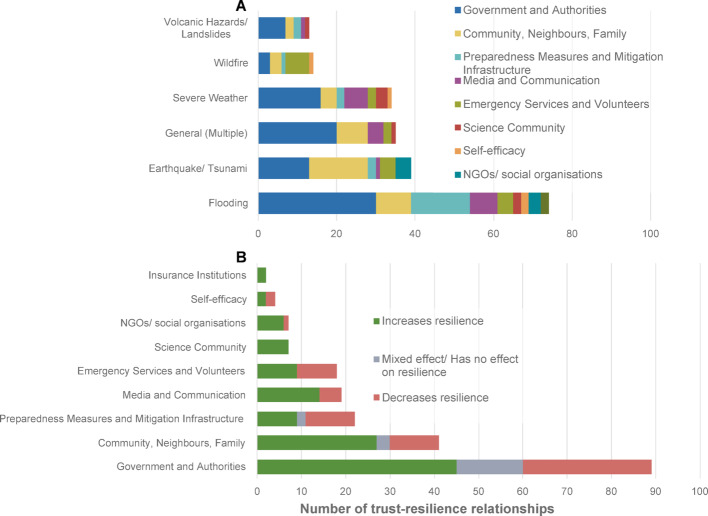
**A** Number of trust-resilience relationships for each studied natural hazard type—the diagram is categorised by "trusted party". **B** Number of trust-resilience relationships per “trusted party” researched—the diagram is categorised by whether trust in that institution was found to increase resilience, decrease resilience, or have no effect/ a mixed effect

Across the 209 relationships, natural hazard types include flooding (35%, n = 74), earthquake/ tsunami (19%, n = 39), general/ multiple hazards (17%, n = 35), severe weather (16%, n = 34), wildfire (7%, n = 14), and volcanic hazards/landslides (6%, n = 13). Across all hazard types except wildfire, the most frequently studied trusted party is the government; but the second most frequently studied trusted party differs systematically (Fig. 5), highlighting the relative importance of different institutions for different hazard contexts. For flooding it is preparedness measures and mitigation infrastructure; for severe weather it is media and communication; for volcanic hazards and landslides it is the science community; and for wildfire it is emergency services and volunteers.

### Relationships between trust and resilience

#### Government and authorities

Trust in government and public authorities is found to be associated with both increases and decreases in resilience, with direction varying by hazard, level of government, and outcome measured. For example, during Cyclone Phailin in India, trusted messages from political leaders raised evacuation compliance, while in Tacloban in the Philippines, pre-existing distrust and credibility concerns around official warnings undermined compliance with official orders (Walch 2018). Similar patterns were observed in surveys following flooding in Germany and the UK where greater distrust in authorities was associated with a greater likelihood of delayed evacuation (Mahdavian et al. 2020).

Research from Yushu, China links high ability-trust in government entities with adoption of preparedness measures—residents trust in the government and will consequently do what the government recommends (Han et al. 2021). However, opposite findings were identified in Wenchuan, China and in Jordan where higher general trust and ability-trust in government respectively correlated with fewer preparatory actions (Han et al. 2017; Gammoh et al. 2023)—here, trust in government is associated with higher perceived safety and a perception of fewer required protective actions. Where trust in government is high, organisations engaged more in disaster-related activities (Huss et al. 2012); homeowners report a higher intention of taking household mitigation actions (Ghasemi et al. 2020); and residents are more willing to pay for and participate in disaster management (Kim et al. 2020; Kim et al. 2025). Conversely, home-buyout programme participation, acceptance of government-approved relocation, and desire for government-community cooperation were all found to fall where distrust in government was high (Schwaller et al. 2021; Ekoh et al. 2023; Nguyen-Trung et al. 2024).

Seebauer and Babcicky (2018) find that intention-trust in the Austrian government is associated with downplaying risk and more wishful thinking among Austrian households. Similarly, Dias et al. (2020) found that increased trust in the French government decreases an individual’s sense of flood vulnerability. They claim that trust is tied to knowledge; those with limited knowledge of a hazard must trust institutions to get information, while those with high hazard knowledge are able to scrutinise the accuracy and applicability of information presented to them (Dias et al. 2020). These results are counter to those in other studies, where governmental trust increases risk perception. For example, in their study of multiple hazards in Chile, Cisternas et al. (2024) found that trust in the government increased risk perception, though only for flooding; trust in government had no effect on earthquake and tsunami risk perceptions nor any influence on preparedness intention.

Trust in government also affects how communities mobilise. Farmers who cooperated with their local governments to join agricultural cooperatives coordinated by their local governments after the 2015–2016 drought were those with high intention-trust in the Vietnamese government; where there was distrust and lack of participation in cooperatives, it was generally due to the government’s previous policies and emergency management track-record (Nguyen-Trung et al. 2024).

#### Community, neighbours, and family

Trust in one’s community or neighbours is often associated with increased preparedness and collective capacity, though effects vary by context and measure. Studies from the US and Taiwan indicate that higher neighbourhood cohesion and increased confidence in communities was associated with a greater number of preparedness actions (Reininger et al. 2013; Wei et al. 2019). Similar patterns were observed in US urban flooding contexts where social capital (including trust in neighbours) was linked to greater uptake of household mitigation (Bixler et al. 2021) and in China where trust (within a broader risk-communication model) significantly increased willingness to engage in preparedness actions (Hu et al. 2024).

Beyond households, community trust predicted community participation in disaster risk reduction (Ma et al. 2022), evacuation-related sharing of shelter and transport during California wildfires (Wong et al. 2021), and organisation of early response and recovery in a Chilean fishing community following the 2010 tsunami (Moreno et al. 2019). In Canada, expectations of mutual aid rose with increased societal trust (Yong et al. 2020), while in Japan trusted peer messages facilitated rapid risk interpretation of disaster warnings for foreign residents (Cadwell 2019). Evidence on whether community trust shifts risk perception is mixed—evidence from China shows that trust in family was associated with lower perceived earthquake probability, while trust in friends was associated with higher perceived consequences (Han et al. 2017).

#### Mitigation infrastructure

Trust in protective measures can both support and undercut resilience across outcomes. On the supportive side, confidence in shelters facilitated evacuation in India during Cyclone Phailin (Walch 2018). Trust in mitigation measures has also been linked to greater household preparedness intentions for floods in China, higher spatial awareness of coastal flood hazard in the US, and stronger public support and willingness to pay for mitigation funding in Europe (Houston et al. 2019; Verlynde et al. 2019; Zhang et al. 2021).

Many studies link ability-trust in infrastructure to reduced risk perception, which can lead to overconfidence (Terpstra 2011; Houston et al. 2019; Cannon et al. 2021; Zhang et al. 2021). In Portugal’s wildfires, confidence in pumps and sprinklers encouraged residents to stay and defend rather than evacuate (Asfaw et al. 2022). Homeowners in Sweden who distrusted the ability of public flood-risk reduction were more likely to implement private measures (Grahn and Jaldell 2019). Also important is trust in the safety of disaster shelters; where residents distrusted evacuation-centre conditions in Tacloban (Philippines) they stayed at home, while in Odisha (India) confidence in shelters facilitated evacuation (Walch 2018). Critically, mitigation infrastructure is intended to reduce risks; if risks are decreased sufficiently, then reduced risk perceptions may be both expected and logical (Wachinger et al. 2013). Because most studies did not assess the protection afforded by infrastructure, observed reductions in individual risk perception could reflect true risk reduction; where protection is effective, lower perceived risk may be expected.

#### Media and communication

Trust in media and the perceived accuracy of communication is generally a predictor of increased resilience. In the USA, ability-trust in local forecasters predicted higher willingness to evacuate during severe weather (Sherman-Morris 2005); trust in communication predicted policy support for post-disaster risk-reduction measures (Greenberg et al. 2014); and ability-trust in flash-flood warnings was a strong predictor of protective action intentions (Morss et al. 2016). In Vietnam, ability-trust in disaster warning systems and media was associated with higher perceived climate-change risk (Le Dang et al. 2014), and ability-trust functioned as a positive mediator from knowledge to household preparedness intentions in New Zealand (McIvor et al. 2009). In Taiwan, strong ability-trust in the media has been found to foster higher mitigation intentions (Lin et al. 2008).

Conversely, where people distrust official warnings, protective intentions are weaker, implying that building trust in the warning content and conduit is critical for timely action (Morss et al. 2016). During the 2011 Japan earthquake, high intention-trust in ad-hoc translations from friends and acquaintances increased comprehension of warnings and informed evacuation procedures among foreign residents in Japan (Cadwell 2019). Rainear and Lin (2021) reported similar findings in that students who trusted the source of natural hazard communication were more likely to adhere to evacuation orders and recommendations, yet were found to have lower perceived risk. Trust in the media was also associated with reduced resilience; in the Philippines, reliance on TV and radio amid inconsistent or unclear messaging discouraged evacuation (Walch 2018), and in a US organisational sample, relying on media as the top information source was associated with lower reported preparedness (Huss et al. 2012).

#### Emergency services and volunteers

Trust in emergency services and volunteers has a mixed effect on resilience. In European flood contexts, intention-trust in volunteer responders increased risk perception and reduced non-protective responses such as denial and wishful thinking (Seebauer and Babcicky 2018). Among earthquake survivors in China, ability-trust in outside helpers was similarly associated with higher perceived consequences of hazard impacts (Han et al. 2021). During response, residents’ distrust in formal services reduced coordination and situational awareness in remote settlements in Europe (Taarup-Esbensen 2022).

Research suggests that distrust in emergency services can reduce evacuation likelihood, especially if people doubt responders’ ability to protect property or believe they can do better themselves (Asfaw et al. 2022). In rural North America, higher ability-trust in local fire departments’ suppression capabilities was associated with lower adoption of mitigation measures (e.g., fire-resistant siding, sprinklers, fuel placement) and lower generator purchase (Stasiewicz and Paveglio 2022). Likewise, among Chinese earthquake survivors, intention-trust in outside helpers correlated with reduced household preparedness but increased perceived consequences (Han et al. 2021). Post-event trajectories also appear sensitive to distrust; Australian bushfire survivors reported that diminished trust in emergency services hindered recovery and psychological wellbeing (McCormack and Sillick 2017).

#### Self-efficacy

Determining whether personal confidence is associated with increased or decreased resilience can be challenging, as it is difficult to distinguish healthy self-efficacy from overconfidence. In Portugal’s Large Arouca Fire, many residents reported confidence in their own ability and distrust of fire management agencies, chose not to evacuate, and stayed to defend; actions that carried substantial risk despite some successful property defences (Asfaw et al. 2022). Similar findings in France suggest that increased certainty and confidence in a person’s own ability decreases their sense of vulnerability to flooding (Dias et al. 2020). In contrast, greater ability-trust in one’s self-efficacy has been linked to stronger preparedness (Gammoh et al. 2023); those who had greater trust in their own ability to respond to floods were more likely to engage in increased preparedness measures, suggesting that confidence in one’s own capacity to manage and respond to natural hazards may incentivise some towards disaster preparedness.

#### Science community

Trust in scientists consistently aligns with stronger preparedness, risk perception, household mitigation, evacuation, and policy engagement. In the US, believing in the Federal Emergency Management Agency’s (FEMA) capabilities is associated with greater willingness to undertake tornado precautions (Choi and Wehde 2020), while in the United Kingdom, trust in regulatory scientific knowledge correlates with increased adoption of household flood defences (Soane et al. 2010). Additionally, trust in disaster‐warning systems and science‐based advisories is associated with higher mitigation intentions in Taiwan (Lin et al. 2008); trust in science-led advisory systems in the Sahel is associated with increased mitigation intentions and the uptake of agrometeorological expert advice (Vignaroli 2017). Meanwhile in New Jersey, trust in climate scientists predicted support for post-Sandy risk-reduction policies (Greenberg et al. 2014) and trust in scientific information and community is described as a prerequisite for successful collaboration and execution of coastal DRR measures in Europe (Martinez et al. 2020). Trust built through close communication among scientists, emergency managers, and communities increased the effectiveness of crisis communication during the Eyjafjallajökull 2010 eruption (Bird et al. 2017).

#### Insurance institutions, NGOs, and social organizations

Across the limited evidence base, trust in insurance institutions, NGOs, and other social organisations is associated with increased resilience. In Vietnam, higher ability-trust in the institution providing the policy (e.g., private firms and NGOs) was associated with greater willingness to subscribe to those insurance options (Reynaud et al. 2018). In qualitative focus groups with urban residents in the northeastern United States, distrust of the institutions around flood insurance (e.g., concerns about cost, coverage, and claims processes) discouraged (re)purchase, suggesting that increased trust could support sustained coverage (Zinda et al. 2021). Another American study found that increased intention-trust in NGOs and community organisations was linked with increased adoption of mitigation and protective actions (Briccetti, Coleman and Taylor, 2025). In India and New Zealand, pre-existing trust in NGO networks enabled rapid resource mobilization, coordination, and support for recovery and reconstruction (Kilby 2008; Stevenson and Conradson 2017).

## Discussion

From the 100 studies reviewed, we found 209 relationships between trust and resilience. Here we synthesise the meanings of ‘trust’ and ‘resilience’ deployed in the DRR literature and discuss their observed patterns in contemporary DRR research. This review shows that trust is neither uniformly beneficial nor uniformly harmful for natural-hazard resilience. Rather, its effects are conditional on (i) what kind of trust is in play (ability vs intention), (ii) who is trusted, (iii) which resilience component is assessed, and (iv) context (hazard, governance, culture). This pattern resonates with the “resilience to what, for whom?” critique (Cutter 2016) and with long-standing geographical work situating risk in uneven vulnerabilities and governance arrangements rather than hazards alone (Wisner et al. 2003; Kelman et al. 2016). Our findings support a shift from treating “trust” as a uniformly desirable stock to analysing which dimensions of trust, in which actors, under which governance conditions, move different resilience outcomes in different directions.

### Patterns in trust and resilience research

#### Trust is predominantly associated with increased resilience

Of the 209 identified relationships, over half link higher trust to increased resilience (58%). In these instances, trust lowers transaction costs for coordination, heightens the credibility of warnings, and amplifies efficacy beliefs; each of which appears to push individuals towards increased protective behaviour. Overwhelmingly, trust catalyses collective action through social capital in that trust within and across groups (bonding, bridging, linking) can enhance cooperation in response, recovery, and reconstruction; crucially, institutional actors that communicate clearly help create trustful ties that sustain resilience gains (Castro-Correa et al. 2020). This also connects with a larger pool of shareable resources during crises as seen following major California wildfires where higher trust in neighbours and strangers significantly increases willingness to share mobility and sheltering resources. Trust can improve the uptake of risk information and increase motivation to mitigate, particularly where intention-trust is high—this pattern is consistent with protection motivation theory in which trusted sources can strengthen coping appraisals (Faryabi et al. 2023). Another important observed pattern is that trust interacts with experience; where recent trauma is absent, citizen–official trust becomes even more decisive for evacuation compliance, reinforcing our claim that who is trusted, and for what (ability vs intention), influences resilience trajectories (Walch 2018).

A third of the relationships show that trust can be associated with decreasing resilience (33%). In settings where risk management is strongly collective, high trust (especially ability-trust) in authorities may rationally shift effort from private stockpiling to reliance on institutional or community capacities; this may reduce perceived risk and worry, thereby suppressing individual preparedness behaviours without necessarily diminishing system resilience (Papagiannaki et al. 2019). A similar observation can be found with overreliance on community-based social support; too much trust in the ability of the community to assist you can decrease risk perceptions and impair household-level preparedness (Babcicky and Seebauer 2020). When high self-efficacy combines with low ability-trust in authorities, trust can reduce resilience by reducing compliance with evacuation orders (Asfaw et al. 2022). These findings suggest that the “trust reduces resilience” pattern often captures a shift in the locus of capacity (from households to institutions/communities) rather than a net loss of resilience.

#### Studies focus on trust in governments and authorities

More than two-fifths (43%) of the reviewed case studies examine the effect of trust in governments/authorities on resilience. This research is split on whether governmental trust is linked with increased natural hazard resilience (50%), decreased resilience (33%), or has a mixed or no effect on resilience (17%). Despite this wide literature, the varied results reveal the complex relationship between governmental trust and resilience.

While experience and hazard knowledge appear to be the two main drivers influencing where people place their trust, cultural factors are integral in determining the actions people will (or won’t) take as a result of that trust. For example, consider the case studies from the USA and China that account for almost half of the reviewed research. In the USA, trust in the federal government is generally lower than trust in the local government; in China, this is flipped, with residents reporting high levels of trust in the central government and lower trust in local officials (Han et al. 2021). Therefore, it is difficult to compare the results of increased trust in local officials in the USA to increased trust in local officials in China, because there are cultural and historical differences that inform the expected base state of these relationships.

There is less research regarding trust in community/neighbours/family (20%), media (9%), the science community (3%), NGOs/social organisations (3%), and insurance institutions (1%); all of which show that trust in these institutions is associated with increased resilience. This suggests that across numerous hazard types and geographical locations, increased trust in these institutions may be a good indicator of individuals having greater natural hazard resilience. One explanation for why trust in these institutions leads to increased resilience may be that individuals have more personal connections in the community with these people than with the government.

Studies regarding trust in preparedness measures and mitigation infrastructure (11%), emergency services and volunteers (9%), and self-efficacy (2%), are split on their findings, with no clear trend as to whether trust is associated with increased or decreased resilience. Interestingly, of the twenty-two case studies regarding mitigation infrastructure, fifteen of them pertain to flood hazards. Flooding is a common hazard where mitigation infrastructure is particularly visible compared with seismic engineering to buildings or severe weather early warning systems. The visibility of flood protection infrastructure biases these results.

#### Preparedness is the most studied factor of resilience

Individual and household preparedness measures are the most frequently measured component of resilience (30%), followed by risk perception (19%), warnings and evacuation (13%), household mitigation (11%), response and collective action (10%), policy engagement (8%), recovery (7%), and insurance uptake (3%). Interestingly, the studies that discuss preparedness do not consistently define what “preparedness” consists of. For the constructivist research, preparedness is often based on participants’ definition of what preparedness means to them for different hazard types (Castro-Correa et al. 2020), whereas positivist research asks questions regarding specific measures such as taking insurance or having an emergency kit (Wei et al. 2019). There was limited discussion in the articles regarding how preparedness measures may differ for different hazard types. Some authors, however, did discuss the difference between actual measures taken and intended measures (Wei et al. 2019; Asfaw et al. 2022). Previous research has focussed on why people do (or don’t) take preparedness actions (Wachinger et al. 2013). Beyond trust, preparedness actions can be influenced by risk perceptions, culture, and capacity (e.g., spending ability). This body of research highlights the complex and interconnected relationship between resilience factors (i.e., increased community support might also influence risk perceptions and risk perceptions might influence preparedness). Our synthesis suggests three primary behavioural pathways through which trust operates.

i. **Trust directly influences resilient actions:** Where intention-trust and ability-trust are both high, people tend to accept warnings, adopt household mitigation, and support DRR policies. This aligns with appraisal models that emphasise credible messengers and coping efficacy (Terpstra 2011). It is also visible in our corpus whenever trust in science communities, media/warning systems, NGOs, or capable local authorities predicts greater preparedness, policy engagement, or evacuation.
ii. **Trust creates an environment of “safety” which results in fewer resilient actions:** Trust can reduce perceived vulnerability sometimes appropriately (because protection works), sometimes not. High ability-trust in mitigation infrastructure frequently lowers risk perception and can displace private action (e.g., fewer household measures behind levees). This is consistent with “levee effect” dynamics and with the “risk perception paradox” (Wachinger et al. 2013), and it explains many of the decreases we observe for government/infrastructure in Figs. 4–5B.
iii. **Trust increases coordination capacity:** Trust embedded in bonding and bridging networks can mobilise mutual aid, information, and rapid collective action, particularly in response and recovery phases. But the same ties can also normalise risky choices (e.g., stay-and-defend in wildfires) or crowd-out evacuation. Thus, community/neighbour trust shows both positive and negative associations across outcomes in our dataset.

Recreancy sharpens these pathways by adding responsibility and accountability. Where institutions are perceived as failing in their obligations, intention-trust erodes and compliance collapses; where competence and procedural justice are visible, trust becomes an enabler of “build back better” trajectories (Tierney 2012, 2025; Ritchie 2024). Cultural cognition helps explain whose messages are deemed legitimate in the first place; congruent messengers (e.g., local leaders, faith organisations) often outperform distant authorities, mediating both compliance and substitution effects.

#### Resilience literature is dominated by hydrometeorological hazards

Hydrometeorological hazards account for almost two thirds of the reviewed case studies with research on flooding, tsunamis, and severe weather. Flooding is one of the deadliest and costliest hazards affecting the planet each year, so it is of little surprise that there should be such a large body of research regarding trust and resilience in a flooding context. However, compared with the other hazard categories, research on flooding has the most evenly split findings on whether trust increased (46%) or decreased (42%) resilience.

These results indicate that resilience is not a fixed attribute that we can assign to an individual or community. It is more likely that resilience is dynamic in space and time and that people have different levels of resilience for different hazard types; this is based on their experience with these hazards, their perception of risk and severity, and their trust in different institutions amongst other factors. People will rely on different systems for the response of different hazards, which may partially explain why trust can increase or decrease resilience for different hazards.

Figure 5A shows the salience of different trust targets by hazard: emergency services dominate wildfire studies, science communities dominate volcanic/landslide settings, media/communication is central for severe weather, and infrastructure/schemes for flooding. This mirrors hazard mechanics: fast-onset hazards (tornadoes, flash floods) hinge on trusted warnings; complex, uncertain, and technical hazards (volcanoes) hinge on trust in scientific interpretation; chronic water risks hinge on confidence in, and calibration of, structural and non-structural measures. The same trusted target can, therefore, legitimately produce opposite behavioural effects across hazards and phases (e.g., “trust in pumps” encourages stay-and-defend in wildfires, but “trust in shelters” increases evacuation in cyclones).

### Differing perspectives of trust and resilience

A central finding is the variability in how researchers define and measure trust and resilience. These differences often stem from contrasting epistemologies. Positivist studies tend to treat both terms as quantifiable, objective constructs (Cannon et al. 2020; Xie et al. 2023), whereas constructivist work emphasises subjective, context-dependent meanings (Dias et al. 2020; Martinez et al. 2020). Consequently, “trust” may refer to confidence in a system’s ability, intention, or both, but survey questions seldom differentiate these dimensions. Likewise, resilience is sometimes framed as the inverse of vulnerability, and other times as one of its components, with trust being only one among several factors (Kelman et al. 2016; Bixler et al. 2021).

Given these varied definitions and measures of trust, there is no single methodology to measure trust and resilience in DRR scholarship. Some researchers propose that trust only matters when people lack hazard knowledge (Dias et al. 2020; Cisternas et al. 2024), highlighting the interplay between expertise and external trust. This is, however, complicated by research which reveals the complexity of what constitutes as "knowledge" and the limitations of formal expertise (Nicholas et al. 2025). Consequently, we do not advocate for strict uniformity but suggest that studies clearly state their theoretical framework, epistemology, and key concepts. We also acknowledge the epistemological limitations of assuming that resilience can be measured at all.

Some studies imply a causal connection, with trust influencing resilience. However, given the complexity of resilience, deterministic language warrants caution; generally the findings imply only an association between the two factors. Some authors note that resilience may also shape trust, thus creating a feedback loop. For instance, individuals protected by a levee (Cannon et al. 2021) or successfully defending property during a wildfire (Asfaw et al. 2022) can develop greater trust in infrastructure or their own capacity. By contrast, negative experiences (or corruption unrelated to hazards) may erode trust in local authorities (Walch 2018). Importantly, in these examples the individuals had a positive experience. When people engage in risky behaviour and do not feel adverse consequences from a hazard event, it may reinforce vulnerable decision-making and inappropriately placed trust. Conversely, when people engage in risky behaviour and experience negative effects as a result, they are more likely to redirect their trust. Our Fig. 1 schema clarifies that individuals in the top-right quadrant (e.g., high ability-, high intention-trust) should support resilience via both compliance and coordination, while the bottom-right (ability without intention) may trigger resistance (e.g., policy opposition, non-participation), and the top-left (intention without ability) risks disillusionment and eventual recreancy judgments about the competency of responsible institutions. Ambiguous measures blur these mechanisms and likely contribute to mixed findings.

### Limitations

This review considered articles and book chapters written in English and published between January 2000 and August 2025. Consequently, the review is likely missing relevant studies published outside of this time range, in other languages, and in other document formats (e.g., grey literature). Additional studies may have been identified if the authors searched for texts beyond the Scopus and Web of Science databases, and if broader search-terms were used (e.g., searching for “confidence”, “credibility”, and “expectations” in addition to “trust”). Although title/ abstract screening was dual-reviewed, full-text review and data extraction were conducted by a single author. To create as comprehensive a corpus of studies as possible, the authors considered studies across a broad epistemological spectrum. A strength of this approach has been to review the varied ways in which “trust” and “resilience” are both defined and measured; however, this inconsistency also limited our ability to directly compare findings. We also decided to recode measures of distrust as their inverted “trust” effect. This was done out of analytic convenience such that there was a standard effect direction between all studies. However, it assumes a symmetry between trust and distrust which may not hold psychologically or behaviourally despite our sample supporting this pattern (e.g., distrust in government yielding increased preparedness does not necessarily mean that trust in government yields decreased preparedness). Our dataset over-represents Asia and North America as well as hydrometeorological hazards, potentially limiting transferability to under-studied regions and hazards.

## Conclusions and recommendations

Through this systematic literature review of the influence of trust on natural hazard resilience, we found that trust is a key factor influencing people’s knowledge, awareness, and disaster actions. This review highlights that trust can both support and undermine natural hazard resilience; variations stem from how trust is defined (ability, intention, or both), who is trusted, the outcome examined, and context. Resilience is sometimes defined as the opposite of vulnerability, and other times as a component of it. Furthermore, there is a wide spectrum of ideas as to what resilience itself is composed of. We found that resilience can be grouped into personal preparedness measures, risk perception, willingness to evacuate, and community support. This list is not exhaustive and there are likely other dimensions of resilience. These factors are also interconnected.

The findings of each article are contextually specific to the culture, geographical location, and political nature of a region as well as the experiences and beliefs of its residents. Future research should clearly define trust (ability, intention, or both) and resilience within the framework of their epistemology. We conclude with five recommendations to enhance conceptual and methodological clarity in future research. By defining trust more explicitly, examining it across multiple institutions, and tracking its evolution over time, DRR can better capture how people cope with, adapt to, and potentially thrive amid various hazards.

1. **Investigate temporal and contextual dynamics of trust:** Most of the research reviewed provides snapshots from a single point in time. Longitudinal and comparative studies are needed to track how trust and resilience evolve before, during, and after hazard events. Understanding these shifts could illuminate the ways in which trust can strengthen or erode over time and influence risk-reduction behaviours. Future studies could research the differences in people’s resilience to different hazard types. This could also include multi-hazard research that looks at resilience to cascading and compounding events, or research considering how resilience differs based on different triggers for the same hazard (e.g., earthquake-triggered landslide versus rain-triggered landslide).
2. **Further our understanding of disaster preparedness:** Because preparedness is a central dimension of resilience, future research on preparedness should look at discrepancies between intended preparedness measures and actual preparedness actions taken over time. This research could study whether intended preparedness does result in a tangible increase in people’s resilience and better understand the barriers people face to transition from intended to actual preparedness.
3. **Clarify definitions of trust:** One of the most significant challenges in this field is the inconsistent use of the term “trust.” Future research in DRR should explicitly differentiate between ability trust (i.e., confidence that an entity can perform effectively) and intention trust (i.e., confidence that an entity will act in the public’s best interest). Clarity in defining and measuring these distinct facets of trust would help resolve contradictory findings and allow researchers to pinpoint more precisely how trust shapes (or is shaped by) resilience.
4. **State theoretical frameworks and epistemologies:** Studies should clearly outline their theoretical underpinnings, whether positivist, constructivist, or mixed-methods, and specify how they conceptualise key terms like trust and resilience. Such transparency would aid comparison across studies and enable inconsistencies to be considered in light of differing epistemological assumptions.
5. **Expand the range of institutions and hazards studied:** Future research should consider more than just trust in government and authorities. Individuals place trust in a multitude of people and systems, and trust in these different institutions leads to different actions. Future efforts should look beyond governmental trust to examine trust in local communities, NGOs, private firms, scientific bodies, and other social actors. Researchers could study both intention and ability trust in each of these systems to better understand how and where an individual, community, or organisation places their trust. Similarly, hazard research should include more diverse contexts (beyond predominantly hydrometeorological events) to capture how trust and resilience operate in relation to different risks.

## Electronic Supplementary Material

Below is the link to the electronic supplementary material.

Supplementary Material 1

Supplementary Material 2
