# Supplementary material for: How trust shapes individual resilience to natural hazards: a systematic review
Source: Nat Hazards (Dordr). 2026 Jan 19;122(2):48. doi: 10.1007/s11069-025-07816-w (PMC12816113; doi:10.1007/s11069-025-07816-w)
Supplement: Supplementary file 1 — Supplementary Material 1 [file 11069_2025_7816_MOESM1_ESM.docx]

Online Resource 1

Table S1. Examples of how trust was measured dependent on the type of trust.

| Trust Type | Question Type | Question(s) | References |
| --- | --- | --- | --- |
| Ability-trust | Survey – 4-point scale | - On a scale from "Never" to "Always", how much do you trust the following groups for information dissemination and response actions in the event of a disaster: municipal government, civil protection local office, and community representatives. | (Hernández Aguilar and Ruiz Rivera, 2016) |
|  | Survey –5-point scale | - How much do you agree with the following statements: - I trust the fire department to put out fires on my property. - I trust state agencies to put out fires on my property. - I trust federal agencies to put out fires on my property. - I trust contracted firefighters to put out fires on my property. | (Stasiewicz and Paveglio, 2022) |
| Intention-trust | Interview | - Describe an event before, during or after the recent fires which either strengthened or diminished your trust in a particular person, organisation or agency. - What were the main factors that made you feel like you could/could not trust them? - What other things contributed to/diminish your trust in them? | (Sharp *et al.*, 2013) |
|  | Survey – 3,4,5-point scales | - Can most individuals can be trusted? (5-point scale) - Can you trust individuals in your neighbourhood? Can you trust strangers? (4-point scale) - Can you trust how others will behave based on how likely a neighbour, police officer, and stranger would return a lost wallet or purse (3-point scale) | (Yong *et al.*, 2020) |
|  | Survey – 4-point scale | - Generally speaking, would you say that most people can be trusted or that you can’t be too careful in dealing with people? | (Reininger *et al.*, 2013) |
| General Trust/ Combination of Ability and Intention | Survey – 10-point scale | - How much do you agree with the following? - I am confident that FEMA will provide effective assistance to me and my community if we experience a natural disaster. - I trust my county and local governments to do what is right for me and my fellow residents in my local area. | (Choi and Wehde, 2020) |
|  | Survey – 5-point scale | - How much do you... - Trust the scientific community to honestly report its findings related to climate change - Trust that the international scientific community understands the science behind global climate change - Trust that the media I rely on communicates to us honestly - Trust our state and local officials understand the implications of global climate change for our region - Trust federal government to manage redevelopment | (Greenberg *et al.*, 2014) |
